# Psychiatry in Qatar

**DOI:** 10.1192/bji.2023.23

**Published:** 2023-11

**Authors:** Mohammed Mohammed, Ibrahim Makki, Suhaila Ghuloum

**Affiliations:** 1 MD, Psychiatry Clinical Fellow, Hamad Medical Corporation, Doha, Qatar; 2 MD, MFM, Psychiatry Clinical Fellow, Hamad Medical Corporation, Doha, Qatar; 3 FRCPsych, Senior Consultant Psychiatrist, Hamad Medical Corporation, Doha, Qatar. Email sghuloum@hamad.qa

**Keywords:** Mental health, Qatar, psychiatry, development, services

## Abstract

The first article written about psychiatric services in Qatar was published in *BJPsych International* in 2006. Since then, the health system in Qatar has undergone significant transformation in the areas of service delivery, research and education. International accreditations are sought in all these fields to emphasise the standard achieved. In this article, we follow up on the mental health services currently available in Qatar, their strengths and the associated challenges.

Qatar is a small sovereign country in the Middle East, known for its vast reserves of natural gas and oil. Its population of just over 2.9 million people is primarily made up of expatriate workers, with a distinct Qatari identity rooted in the country's unique history and culture, heavily influenced by Islamic teachings. Hospitality and food are important aspects of Qatari culture.^1^ This was shared with the world as the country hosted the 2022 FIFA World Cup. Qatar also targets excellence in health, education and research. Several international colleges and universities have opened camps in Qatar, many under the umbrella of the Qatar Foundation for Science and Technology, which has also launched the largest research fund in the region through its National Priorities Research Program. Hamad Medical Corporation (HMC) provides the largest public healthcare services, including mental health services.

## Historical background

Psychiatry in Qatar has a relatively short history compared with other medical specialties. Traditional healing practices had a significant role in the past, and modern psychiatric care was introduced only shortly after independence in the early 1970s.^2^ Since then, there has been a steady progression of services to meet international standards for mental healthcare delivery. Although traditional healing remains a choice for many, there is an increasing awareness of the importance of evidence-based approaches to mental health.

The Ministry of Public Health in Qatar has worked closely with the World Health Organization (WHO) and other international advisors to support efforts to uplift the standard of mental health service delivery and training. The first Qatar National Mental Health Strategy (2013–2018) was subsequently launched. This was a 5-year strategy with objectives aligned with the WHO Mental Health Global Action Plan. The strategy later developed into the National Mental Health & Wellbeing Strategic Framework 2019–2022 and the broader Qatar National Health Strategy 2018–2022.^3^

The Qatari government has launched various initiatives, including public education campaigns and training programmes for healthcare professionals. In addition, Qatar has become a hub for mental health research and innovation.

## Service provision

Mental healthcare in Qatar is provided by the mental health services of HMC with minimal privately run clinics. The National Mental Health Strategy called for the more prevalent disorders to be treated at the primary care level, while HMC focused on the less prevalent and more severe disorders. The Primary Health Care Corporation thus provided extensive training for many general practitioners and family physicians in the management of anxiety and depression. Guidelines and referral policies were developed to facilitate incorporating mental health into primary care, albeit with some limitations.

The provision of mental health services therefore remains primarily led by the psychiatry department of HMC. The services are accredited by Joint Commission International as an academic facility. Currently, there are 106 acute in-patient beds; together with 28 residential beds, these represent the only in-patient service for the entire population. Subspecialties have gradually expanded, and community mental healthcare has been broadened to cover a wider geographical distribution.

The department works in collaboration with other services providing direct or indirect mental healthcare in the country. Outreach is provided to college campuses and to Qatar Airways. Sidra Hospital provides tertiary healthcare for women and children. The women's psychiatry care at Sidra was initially limited to the peripartum period and has only recently been expanded, on a private basis, to general adult mental health.

Addiction services are managed separately. A centre for treatment of addictions and behavioural disturbances, Alawain, was later replaced with a specialised addiction treatment and rehabilitation facility, Naufar. The scope of service for Naufar necessitates voluntary admissions, with minimal allowance for legally enforced treatment. Subsequently, to meet national demands, a new service was opened in 2022. The latter is run by mental health services, with admissions only possible through a general prosecutor's order.

With the breakout of the COVID-19 pandemic, there was an urgent need to address anticipated rising demands for mental healthcare while ensuring minimal interruption of services for those who need them. A national mental health helpline was developed early in the pandemic to provide psychology, nursing and psychiatric care, and the scope of its work was gradually expanded to a 24/7 basis. The urgent need to introduce telepsychiatry prompted another rapid addition to services. This was met with higher acceptance among the public and a lower no-show rate compared with out-patient clinics.^4,5^

## Education and training

There was initially only a nursing school available locally, and Qatari citizens wishing to study medicine were sponsored to study abroad, regionally or internationally. Weill Cornell Medicine-Qatar was the first medical school to open in Qatar, associated with Weill Cornell Medicine in New York, USA. The College of Medicine at Qatar University opened later in 2015. Locally, there are currently provisions for studying psychology and nursing. Other allied health professionals are trained or recruited internationally.

Whereas the residency training programme once relied heavily on international applicants, graduates of the two local medical schools now form the majority of trainees in psychiatry. The Psychiatry Residency Training Program at HMC is a 4-year programme accredited by the Accreditation Council for Graduate Medical Education International. Fellowship programmes have been developed over the past few years, providing further and higher training in subspecialties of psychiatry. These are locally accredited.

Licensing for medical specialties and allied health has gradually developed and become mandatory. Many opportunities are available to locally obtain continuous professional development credits and support for attendance at international conferences.

## Medical research

Qatar is rapidly expanding its medical research field to become a regional centre, supported by the Qatar National Research Fund, which offers funding programmes such as the National Priorities Research Program and the Undergraduate Research Experience Program. The Qatar Biomedical Research Institute and Sidra Medicine Research Department are actively involved in developing diagnostics and therapeutics for prevalent diseases in Qatar and the region. In addition, Qatar hosts international collaborations including the Qatar Genome Programme and the Qatar Cardiovascular Research Center. Qatar is also a hub for hosting medical conferences and events such as the World Innovation Summit for Health. The infrastructure for research has also improved at colleges and hospitals, with concomitant increases in allocated research budgets.

## Prospects and challenges for mental health in Qatar

Qatar has made significant progress in recent years in expanding access to healthcare, including mental health services. However, there are still challenges that need to be addressed in order to ensure that people have access to the care and support they need to maintain good mental health.

One of the major strengths for mental health in Qatar is the government's commitment to prevention, raising public awareness of mental health issues and providing support for those in need. The Ministry of Public Health has launched a number of initiatives to promote mental health and well-being, including awareness campaigns and the establishment of mental health clinics in hospitals throughout the country. Clinically, we observe more readiness to seek psychiatric help, especially among the young. However, this has not been researched formally.

Another strength is the growing availability of telepsychiatry services in Qatar.^4^ With the pandemic forcing many people to stay at home and limiting access to in-person care, telepsychiatry services had to be introduced urgently and have become an increasingly popular option for many patients, especially in the context of high stigma in this part of the world.

Despite notable progress over the years and growing prospects for mental health in Qatar, there are still challenges that need to be addressed to ensure that everyone has access to the mental healthcare and support they need, at the highest standard and at the right place and time. Stigma associated with mental illness remains a major challenge. Although efforts to reduce it are ongoing, the impact of these efforts has not been formally measured. Traditional healing remains a viable option for many, and is often the first option before seeking professional help.

Another challenge is the need to expand mental health services to semi-rural areas of the country. Although mental health clinics have been established in hospitals throughout Qatar, there is still a shortage of mental healthcare providers, doctors, nurses and allied health professionals. This can make it difficult for people living in certain areas to access the care and support they need.

The workforce continues to rely heavily on international recruitment. The two medical schools currently provide most of the medical trainees recruited into the psychiatry residency training programme. In more recent years, psychology graduates from Qatar University have provided the bulk of the psychologists’ workforce. International consultancies have been engaged to provide them with clinical training to prepare them for practice. Supervision is an ongoing challenge. The master's programme in clinical psychology of the Doha Institute for Graduate Studies also contributes to the psychologists’ workforce. However, local demand for nurses and other allied health professionals can only be met internationally. Such recruitment has its own challenges, with varying standards of training and practice, language limitations and questions of employment stability.

Qatar's first mental health law was launched in 2016, although it has still not been implemented. Balancing the rights of patients with mental illness with the expectation that society will be protected from harm, and helping families to manage relatives with severe, recurrent or treatment-resistant conditions are difficult tasks in a collectivist society.

In conclusion, Qatar has made significant progress in improving access to mental health services across the lifespan, with growing numbers of subspecialties and expanding geographical distribution of services. Although there are still challenges to be addressed, the government's commitment to promoting mental health and well-being supports further aspirations for the future.
